# Prenatal Maternal Cortisol Levels and Infant Birth Weight in a Predominately Low-Income Hispanic Cohort

**DOI:** 10.3390/ijerph17186896

**Published:** 2020-09-21

**Authors:** Alicia K. Peterson, Claudia M. Toledo-Corral, Thomas A. Chavez, Christine H. Naya, Mark Johnson, Sandrah P. Eckel, Deborah Lerner, Brendan H. Grubbs, Shohreh F. Farzan, Genevieve F. Dunton, Theresa M. Bastain, Carrie V. Breton

**Affiliations:** 1 Department of Preventive Medicine, Keck School of Medicine, University of Southern California, Los Angeles, CA 90032, USA; aliciap@usc.edu (A.K.P.); claudia.toledo-corral@csun.edu (C.M.T.-C.); tachavez@usc.edu (T.A.C.); naya@usc.edu (C.H.N.); markj939@usc.edu (M.J.); eckel@usc.edu (S.P.E.); sffarzan@usc.edu (S.F.F.); dunton@usc.edu (G.F.D.); bastain@usc.edu (T.M.B.); 2 Department of Health Sciences, California State University Northridge, Northridge, CA 91330, USA; 3 Eisner Health, Los Angeles, CA 90015, USA; dlerner@eisnerhealth.org; 4 Department of Obstetrics and Gynecology, Keck School of Medicine, University of Southern California, Los Angeles, CA 90033, USA; brendan.grubbs@med.usc.edu; 5 Department of Psychology, University of Southern California, Los Angeles, CA 90089, USA

**Keywords:** cortisol, birth weight, pregnancy, stress

## Abstract

Infant birth weight influences numerous health outcomes throughout the life course including childhood obesity and metabolic morbidities. Maternal experience of stress, both before and during pregnancy, has been hypothesized to influence fetal growth and birth outcomes. However, these associations currently are not fully understood, due to conflicting results in the published literature. Salivary cortisol is often used as a biological biomarker to assess the diurnal pattern of the hypothalamic–pituitary–adrenal axis (HPA-axis) functioning. Cortisol metrics include both the total cortisol concentration secreted during waking hours, reflected by the area under the curve (AUC), and cortisol dynamics, which include the diurnal cortisol slope (DCS) and the cortisol awakening response (CAR). This study examined the association of these cortisol metrics measured during the third trimester of pregnancy and infant birth weight among 240 mother-infant dyads participating in the Maternal and Developmental Risks from Environmental and Social Stressors (MADRES) pregnancy cohort study, which is predominately comprised of Hispanic low-income women. There were no significant associations with the maternal biological stress response and infant birth weight in this study. More research is needed in larger studies to better understand how the biological stress response influences birth weight in populations facing health disparities.

## 1. Introduction

Infant birth weight is an indicator of the intrauterine environment and is associated with numerous health outcomes throughout the life course [1,2]. Low birth weight, defined as <2500 g, increases the likelihood of childhood obesity and subsequent metabolic health morbidities, such as type 2 diabetes [3,4,5,6,7]. Fetal macrosomia (often defined as ≥4000 g) reflects infants large for gestational age at birth and can also influence childhood weight gain and obesity risk [8,9,10]. Numerous maternal characteristics may positively or negatively impact infant birth weight, including smoking, nutrition, and hypertension [11]. In addition to these known risk factors, maternal experience of stressful life events and the associated biological stress response, both before and during pregnancy, have long been hypothesized to influence fetal growth and birth outcomes [12,13,14,15,16].

Health disparities exist between racial/ethnic groups as well as income levels [17]. Income levels below the poverty line during pregnancy have been shown to be associated with negative birth outcomes due to decreased access to care as well as factors related to maternal stress [18]. Within the United States, Black and Hispanic populations experience higher rates of preterm birth and low birth weight compared to their non-Hispanic White counterparts [19]. Minority groups face unique stressors including limited cultural sensitivity, discrimination, and acculturative stress for immigrants such as separation from family, change in traditions, and language barriers [20,21]. These stressors are hypothesized to affect overall health in many ways. Given the predictive value of birth weight on health outcomes throughout the life-course [1], it is important to understand how prenatal stress and the associated biological stress response influence birth weight in populations facing health disparities.

Cortisol is a key hormone that is produced by the hypothalamic–pituitary–adrenal axis (HPA-axis), which is one of the major components of the physiological stress response system. Cortisol secretion follows a circadian pattern, with daily cortisol levels rising in the hours prior to awakening with a peak approximately 30 min after awakening, followed by a decline throughout the day and reaching the lowest levels in the late evening hours [22,23,24]. Although the diurnal pattern remains consistent during pregnancy, a woman’s baseline cortisol secretion increases up to four-fold compared to non-pregnancy levels, with the highest levels in the third trimester in preparation for birth [25,26]. In addition to this natural increase needed to support fetal development, daily stressors (physiological and psychological) can cause sharp short-term fluctuations of cortisol levels [27,28]. While in utero, the placental enzyme 11β-hydroxysteroid dehydrogenase type 2 (11β-HSD2) keeps the fetus largely blocked from maternal cortisol by converting it to an inactive form. Although 11β-HSD2 blocks most of the maternal cortisol, a small proportion of maternal total cortisol passes through the placenta to the fetus. This exposure has been related to shorter gestation and negative effects on fetal development, including lower birth weight, by reducing the blood flow needed for oxygen and nutrients [29,30,31,32,33]. Additionally, prenatal corticosteroids repeatedly prescribed to women at risk for preterm birth have shown to influence poor fetal growth and lower birth weight [34]. Prenatal studies on the effects of HPA-axis on infant birth weight have produced mixed results. Although higher prenatal cortisol concentration has been identified as an independent predictor of lower infant birth weight in some study populations [35,36,37,38,39,40,41,42,43], it has not been shown in others [44,45,46]. This divergence in results may be due to differing demographics within study populations and their stress responses, or inconsistencies in the methods used to capture and interpret cortisol concentrations and diurnal responses [47].

In order to further elucidate the relationship between maternal HPA-axis activity during pregnancy and fetal growth, we aimed to explore the association of the maternal biological stress response during the third trimester through several cortisol metrics. These metrics include the total cortisol concentration secreted during waking hours, reflected by the area under the curve with respect to ground (AUCg), and cortisol dynamics, which includes both the diurnal cortisol slope (DCS) and the cortisol awakening response (CAR). The DCS reflects the change in cortisol secretion from morning to evening while the CAR represents the increase in cortisol secretion specifically in the 30–45 min from awakening [48,49]. These cortisol metrics (AUCg, CAR, and DCS) during the third trimester of pregnancy were assessed for their influence on infant birth weight in a predominately low-income Hispanic pregnancy cohort.

## 2. Materials and Methods

### 2.1. Sample

All participants were enrolled in the ongoing maternal and developmental risks from Environmental and Social Stressors (MADRES) pregnancy cohort study. The cohort is based in urban Los Angeles and is predominately comprised of Hispanic women, followed by a smaller sub-set of Black women. The majority of mothers are of low-income, primarily insured by Medicaid/Medi-Cal, which are strong indicators of health inequities. Methods of the MADRES study and protocol have been described previously [50]. In brief, participants were recruited during pregnancy from four prenatal clinic sites. These include two community health clinics, one county hospital prenatal clinic, and one private obstetrics and gynecology practice. Eligibility for participants at the time of recruitment included: (1) less than 30 weeks pregnant, (2) at least 18 years of age, and (3) a fluent speaker of English or Spanish. Exclusion criteria for the study included: (1) multiple gestation; (2) having a physical, mental, or cognitive disability that would prevent participation or ability to provide consent; (3) current incarceration; and (4) HIV positive status. At study entry, informed consent was obtained from each participant, and all study aspects were approved by the University of Southern California’s Institutional Review Board.

**Figure 1:**
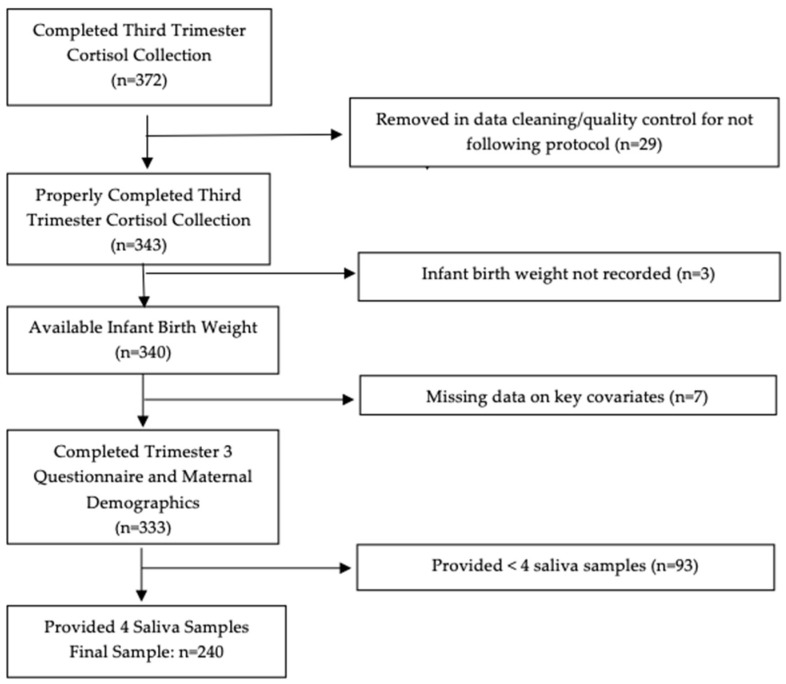
Consort diagram of included mother–infant dyads.

Within the MADRES cohort, 372 mothers completed at-home saliva collections over one day (up to four samples) in their third trimester of pregnancy (sample range: 26–35 weeks gestation). A total of 240 mother–infant dyads with complete maternal cortisol data, birth outcomes data, and key maternal and infant covariates were included in the current analysis. After cleaning the raw data (*N* = 372) and completing quality control checks to ensure all samples were taken on the same day by individually looking at each mother’s reported times and dates, 343 mothers collected at least one sample according to the protocol (explained in more detail below). Three dyads were then removed for not having recorded birth weight available, seven dyads were removed for missing maternal demographic data and/or key covariates collected in the third trimester questionnaire, and 93 were removed for providing less than four saliva samples on the assessment day, which were needed to calculate the cortisol concentration of AUCg. The consort diagram presenting sample exclusions are shown in Figure 1. Maternal and infant demographic and health characteristics were similar (data not shown) in mothers who properly provided one or more saliva samples across the day (*N* = 343) to those who completed collection of all four samples across the day (*N* = 240).

### 2.2. Salivary Cortisol Collection

In order to measure the diurnal pattern of cortisol secretion, participants completed an at-home, non-invasive, salivary cortisol collection using a Salivette device (Sarstedtf, Inc. Rommelsdolf, Germany) over a one-day period in their third trimester of pregnancy (mean gestational age at collection 30.8 ± 2.0 weeks). Most participants completed this sample collection on a weekday (84.6%). Participants were instructed to gently chew on a cotton dental roll for two minutes at time of awakening, thirty minutes after awakening, in the afternoon (between 3–4 pm), and before bedtime. Participants were educated on the importance of following the protocol, and they were instructed to record the exact time and date that each sample was completed on the collection tube. The participants were also asked to mark if they ingested anything other than water, smoked, brushed their teeth, or exercised within the 30 min prior to collecting the sample. Non-adherence to this was flagged for possible contamination. These instructions were provided over the phone with the participant. In addition, each participant was also given a pamphlet with detailed yet easy to understand directions and pictures for each step. This pamphlet also included the contact information to a study staff member if any additional questions arose while completing the saliva collections.

Within the final study sample (*n* = 240), 91.7% of participants completed the first sample within 15 min of waking up and 93.75% completed the second sample within 15–45 min of the first, reflecting high compliance. The collection time with the least reported contamination was for the awakening sample (2.5%), while the highest was seen in the afternoon sample (34.6%), mostly due to eating or drinking prior to collection. The samples that were outside of the instructed times, possibly contaminated, or larger than four standard deviations (2.8%) were flagged for additional sensitivity analyses.

After participants collected each saliva sample, they were instructed to store them in their refrigerator until the day of their third trimester visit (mean time between collection and visit = 5.5 ± 8.8 days). Saliva samples were then stored in a laboratory freezer at −80ºCelsius until they were sent to a commercial laboratory to be assayed for cortisol. They were then frozen and stored at −20ºCelsius until analysis. After thawing, Salivettes were centrifuged at 3000 rpm for five minutes, which resulted in a clear supernatant of low viscosity. Salivary concentrations were measured using commercially available chemiluminescence immunoassay with high sensitivity (IBL International, Hamburg, Germany). The intra-assay and inter-assay coefficients for cortisol were below 9%.

Cortisol levels measured in nanomoles per liter (nmol/L) for each time point were then converted into total cortisol concentration during waking hours reflected by the AUCg and cortisol dynamic variables (CAR, DCS). The AUCg was calculated using the standard trapezoidal formula, which requires all four samples to be complete and considers the time between sample collection and the cortisol level at each collection [51]. Within those who had four complete salivary samples, the CAR was calculated, which represents the change in cortisol levels from the awakening sample and the 30 min post awakening sample, while also incorporating the time between each sample [24,52]. In addition, the DCS was also calculated, which includes the change in cortisol levels and time between the awakening sample and the bedtime sample [53]. Neither non-adherence to the morning collection protocol nor sample contamination significantly altered the AUCg, CAR, or DCS results. In addition, no significant differences were seen in the AUCg, CAR, or DCS across participants who completed the sample on a weekday compared to a weekend (data not shown).

### 2.3. Outcome

For the majority of participants (97.5%), infant birth weight was directly abstracted from the electronic medical records (EMR). A small percentage of participants (1.7%) did not have physician measurements of birth weight recorded on the EMR but had a proxy report (mother) of birth weight noted on the EMR. The remainder of participants (<1%) did not have any birth weight recorded on the EMR, and birth weight was obtained from the mother via an interviewer-administered questionnaire with a MADRES staff member 7–14 days post-birth.

### 2.4. Covariates

Potential covariates were identified a priori based on previous literature and included factors related to study design, maternal demographics, and pregnancy and birth outcome variables. Preliminary analyses were then conducted for their univariate associations with the cortisol metrics (AUCg, DCS, and CAR) and birth weight. Study design variables included recruitment study site and gestational age (in weeks) at time of saliva sample collection.

The maternal demographic covariates included: race, ethnicity (Hispanic vs. non-Hispanic), education level, annual household income, maternal age at baseline, personal smoking status during pregnancy, gestational diabetes status, preeclampsia status, and pre-pregnancy BMI (BMI; kg/ m^2^). These variables were self-reported via interviewer-administered questionnaires in English or Spanish. The only exceptions were gestational diabetes status and preeclampsia status, which were both abstracted from the EMR (Yes vs. Not-Recorded), and pre-pregnancy BMI. Pre-pregnancy BMI was calculated using self-reported pre-pregnancy weight and standing height at the first study visit (<30 weeks gestation) measured by a study staff member via stadiometer (Perspectives enterprises model PE-AIM-101), or height from the EMR if missing. We did not use measured weight at the first study visit due to the range in weeks of gestation upon study enrollment.

The pregnancy and birth-related covariates included: gestational age at birth (in weeks), delivery type (normal spontaneous vaginal, planned cesarean section, unplanned/emergency cesarean section, vaginal birth after cesarean, vacuum-assisted vaginal, and forceps-assisted vaginal), newborn sex, parity, and nausea experienced during the third trimester of pregnancy. Gestational age at birth was calculated and standardized using a hierarchy of methods [54]. A first trimester (<14 weeks gestation) ultrasound measurement of crown-rump length was considered ideal and was used if available (*N* = 142). If unavailable, a second trimester (<28 weeks gestation) ultrasound measurement of fetal biparietal diameter was used (*N* = 68). If measurements from an early ultrasound were unavailable, gestational age at birth was calculated based on a physician’s best clinical estimate from EMR (*N* = 30). Delivery type and infant sex were abstracted from EMR; if these variables were missing from the maternal medical records, mode of delivery and infant sex were obtained via interviewer-administered questionnaires in English or Spanish 7–14 days after birth. Parity was self-reported via interviewer-administered questionnaire at the first study visit. Mother’s nausea was self-reported via interviewer-administered questionnaire during the third trimester in English or Spanish using the Pregnancy-Unique Quantification of Emesis (PUQE) scoring system. This scoring system enumerates the severity of nausea and vomiting during the preceding 24 h on a scale from 3 to 15. This was then dichotomized to mild nausea and vomiting (scores ≤ 6, 95.8%) and moderate nausea and vomiting (scores 7–12, 4.2%), as we had no mothers with severe scores (scores ≥13) [55].

### 2.5. Statistical Analysis

AUCg was right-skewed; therefore, values were log-transformed to decrease the influence of extreme values and to meet the linearity and homoscedasticity assumptions needed for linear regression. Analyses for DCS and CAR were performed on the original scale as these models met the assumptions of linear regression. Delivery type was dichotomized to vaginal or cesarean birth, and parity was modeled as first-born, second-born, third-born, or fourth-or-more-born.

We assessed the univariate relationships between all potential covariates with cortisol metrics (AUCg, DCS, CAR) and infant birth weight. Pearson correlations were used with continuous covariates, which included GA at birth, GA at sample, maternal age, and pre-pregnancy BMI. Independent Student *T*-Tests were used with dichotomous covariates, which included infant sex, delivery type, personal smoking status, gestational diabetes status, preeclampsia status, PUQE nausea and vomiting, and ethnicity. Analysis of variance (ANOVA) was used with categorical covariates, which included race, parity, education level, and income level.

Within all multivariable models, infant sex, GA, maternal age, and ethnicity were deemed necessary due to their a priori importance in similar studies, as well as recruitment site as it is a crucial study design variable within MADRES. Confounding was then assessed by a beta estimate change larger than 10% for each individual stress metric by a one-by-one variable approach for all remaining covariates via linear regression. Confounding was observed by pre-pregnancy BMI and parity.

After assessing the influence of a priori and possible covariates with stress metrics (AUC, CAR, and DCS) and infant birth weight, the following variables were included in all multiple linear regression models: GA at birth, parity, continuous pre-pregnancy BMI, infant sex, maternal age at study entry, ethnicity, and study recruitment site. The selected covariates were then used in multiple linear regressions in three independent models, one for each cortisol metric variable (AUC, DCS, CAR). Sensitivity analyses were then conducted to determine whether results were similar after excluding preterm births (<37 weeks gestation). The significance level for analyses was set at an alpha of 0.05. All data were analyzed using SAS v9.4 (SAS Institute, Inc., Cary, NC, USA).

## 3. Results

### 3.1. Participant Characteristics

Maternal and infant characteristics are presented in Table 1. Overall, the mothers were on average 29 ± 6 years of age, 81% were Hispanic, the majority (56%) had a high school diploma or less, and they had a mean pre-pregnancy BMI of 29 ± 7 kg/m^2^. Their infants were born at a mean of 39 ± 1.5 weeks gestation and weighed on average 3302 ± 497 g at birth. The majority of infants were the first-born (35%) or second-born child (28%), and most were born vaginally (75%). Maternal smoking was not considered to influence birth weight in this sample, as the number of mothers who smoked at any point during the pregnancy was low (*n* = 5, 2.1%).

### 3.2. Biological Stress Measures

**Figure 2:**
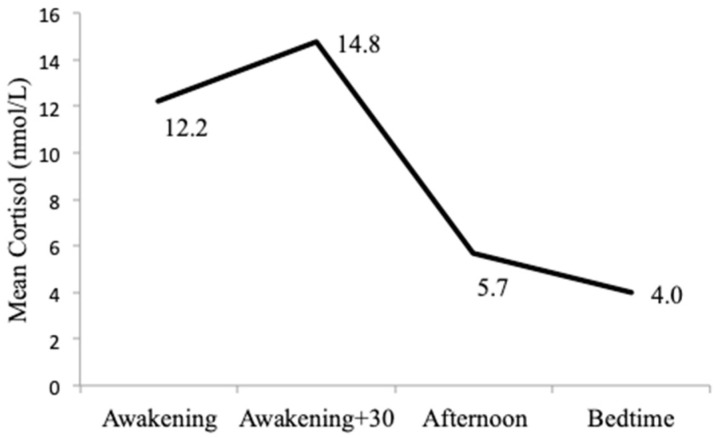
Mean cortisol values for 24 h collection.

**Figure 3:**
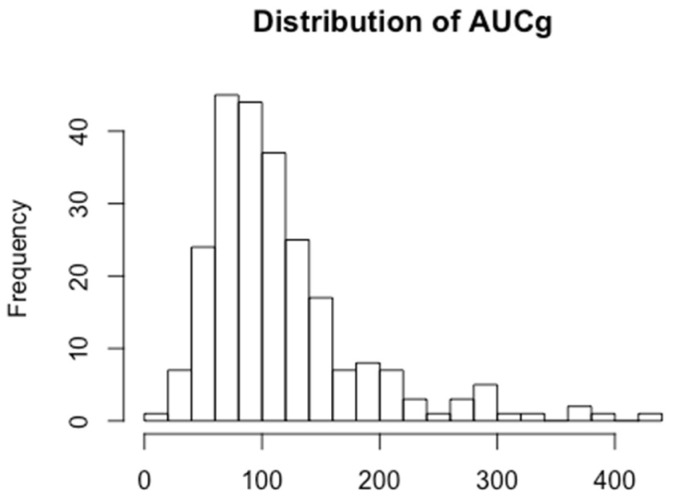
Area under the curve, grounded distribution.

The four salivary cortisol samples had the following mean concentrations: awakening sample 12.2 nmol/L, awakening +30 min sample 14.8 nmol/L, afternoon sample 5.7 nmol/L, and bedtime sample 4.0 nmol/L. These mean concentrations from the four salivary cortisol samples followed the expected daily circadian pattern of the HPA-axis and are shown in Figure 2. Transformation of these values into cortisol concentration (AUCg) and dynamic variables (CAR, DCS) are shown in Table 2. The distribution of AUCg is shown in Figure 3.

### 3.3. Biological Stress Measures with Infant Birth Weight

The results of the three adjusted cortisol metric models with infant birth weight are shown in Table 3. No significant associations between cortisol concentration (AUCg) or cortisol dynamics (DCS, CAR) were seen with infant birth weight. Although not significant (*p* = 0.25), log-transformed AUCg had an inverse relationship with infant birth weight after adjustment for covariates (*B* = −5.6 g for each 10% increase in non-logged AUCg). DCS had a positive relationship with infant birth weight after adjustment for covariates (*β* = 78.3); however, the association was not statistically significant (*p* = 0.08). CAR had no relationship with infant birth weight after adjustment (*β* = −0.4, *p* = 0.98). We tested whether the relationship between prenatal cortisol measures and infant birth weight was sensitive to babies born prematurely (<37 weeks gestation); however, our results were similar after excluding pre-term births in the analysis (Table A1).

## 4. Discussion

Within the United States, Black and Hispanic populations experience higher rates of preterm birth and low birth weight compared to their non-Hispanic White counterparts [19]. Given the predictive value of birth weight on health outcomes throughout the life-course [1], it is important to understand how prenatal stressors influence birth weight in health disparity populations. Existing literature on this relationship is still unclear. In this study of predominately low-income Hispanic women, we found no statistically significant associations between the maternal biological stress response, measured by salivary cortisol concentration (AUCg) and diurnal cortisol dynamics (DCS and CAR) during the third trimester of pregnancy, with infant birth weight. Although not significant, an inverse relationship was seen between AUCg and birth weight while a less steep or more blunted diurnal slope was seen to increase birth weight. These results suggest a trend that should be explored further.

Cortisol levels are often measured using saliva samples taken throughout the day as a biological biomarker of HPA-axis activity, where the overall cortisol concentration during waking hours is reflected by the AUCg [22,51]. Cortisol exposure can also be assessed by the cortisol dynamics of the CAR [24,49,52], which considers the difference in cortisol levels from awakening to the peak 30 min after awakening, as well as the DCS, which reflects the change in cortisol levels from morning to evening [53]. With regard to the literature on HPA-axis activity and birth weight, the reported methodologies associated with each of these aggregate measures varies by study design, making valid comparisons of results between different studies challenging. Beyond differences in trimester of collection, which affects natural levels of cortisol, methodological differences include differing times of day and number of collections required by participants. This is especially sensitive when comparing the CAR across studies.

Results within the literature on salivary prenatal cortisol and infant birth weight are mixed. A recent review by Cherak et al. concluded there was a consistent negative association between prenatal maternal cortisol levels and infant birth weight. This review also observed a substantial heterogeneity in effects by correlation coefficients indicating the likelihood of publication bias. This publication bias was indicated by the uneven distributions of studies and the strongly negative correlations possibly influenced by small study biases. This was seen in overall pooled results, but especially in studies that assessed cortisol during the second and third trimesters [47].

Most of the studies published on the topic have been conducted predominately in White and highly educated populations [35,36,38,39,41,42,46]. Only five studies have been completed in diverse populations similar to the MADRES cohort both ethnically and socio-economically. Within these studies, most used the CAR [40,44,45] or the diurnal slope [37,40,45] to measure the stress response, while one (a cohort of pregnant Hispanic adolescents [43]) used the AUC. Overall, there were no consistent patterns of significant associations across these five studies. Two studies reported null results between maternal cortisol and birth weight [44,45]. D’Anna-Hernandez et al. observed a significant inverse correlation between diurnal slope and birth weight in late pregnancy (34 ± 1.4 weeks gestation) but not in early (17.3 ± 1.8 weeks gestation) or mid pregnancy (28.1 ± 1.5 weeks gestation) [37], and Guardino et al. observed a significant inverse correlation of diurnal slope with birth weight, but not with the CAR [40]. Within the study of pregnant Hispanic adolescents, Spicer et al. found a significant inverse relationship with the AUC and birth weight when outliers were removed, but results were insignificant when outliers were included or when missing values were imputed [43]. Some of the possible reasons for the observed heterogeneity may be due to variations in study methods by number of days, times of day, and trimesters of cortisol collection as well as which cortisol measures were used in analyses (AUC vs. CAR vs. DCS). Overall, our current study contributes additional evidence that there is a lack of significant associations between maternal biological stress response during pregnancy and infant birth weight in a predominately Hispanic and low-income pregnancy cohort.

Although no significant associations were seen in this analysis, it is important to note that the beta estimates for the AUCg and DCS with birth weight were not close to zero. An inverse relationship was seen between AUCg and birth weight. This is in line with what Spicer et al. observed (*β* = −0.02, *p* = 0.03, with outliers removed). However, that study averaged cortisol levels taken from one day over two trimesters in pregnancy and was in a Hispanic adolescent population, which may make results not fully comparable [43]. Conversely, we observed a positive relationship between prenatal DCS and birth weight, indicating a less steep slope in the diurnal pattern, which is commonly associated with maladaptation of the biological stress response with higher infant birth weight [56]. Bublitz et al. additionally found no statistically significant association with diurnal slope and birth weight (*p* > 0.06), although the direction was not reported [45]. In contrast, D’Anna-Hernandez et al. found a significant correlation in the opposite direction to which we observed (*r* = −0.29, *p* = 0.05), with lower infant birth weight being related to a blunted cortisol slope in late pregnancy (34±1.4 weeks gestation); however, this association was seen in weeks later than our study and was insignificant (*r* = −0.003, *p* = 0.97) in weeks closer to our collection (28.1 ± 1.5 weeks gestation) [37].

Many strengths are found within the current study. This study measured salivary cortisol over four time points throughout the day, which allowed us to examine various cortisol metrics (AUCg, DCS, and CAR) to evaluate the association of the biological stress response with infant birth weight in a predominately Hispanic population. Evaluating the biological stress response in a population that is underrepresented in research and facing unique stressors as well as health disparities is an additional strength. This study was also conducted with a larger sample size relative to the literature, especially when compared to those with predominately Hispanic populations (D’Anna-Hernandez et al. *n* = 55 and Spicer et al. *n* = 119) [37,43].

Limitations of the study include that participants completed cortisol collection at home and were responsible for marking the time of the sample, which could potentially introduce exposure misclassification. Assessing accurate cortisol patterns through salivary collection requires high compliance and motivation from study participants, which may be especially challenging in this population facing unique stressors. Raw data were vigorously checked for participant errors, lab errors, and data entry errors, and quality control steps were taken to reduce these errors as much as possible. It has additionally been suggested that at least two days of cortisol collection consisting of 3–6 samples per day are needed to appropriately capture the activity of the HPA-axis [57]. It has also been advised that three morning samples should be used to calculate the CAR, rather than two on any given day [49]. However, both these guidelines increase the burden on study participants [57] and were infeasible in our study population. The possibility of self-selection bias is also present. The pregnant mothers who completed the cortisol collection could have been less stressed, therefore having more time or motivation to complete the four samples than the ones who chose not to participate. Of all MADRES participants (*N* = 843), 62% of pregnant mothers came in for their third trimester visit (*N* = 519). Demographics of these 62% of participants are similar to the overall cohort. Of the 519 mothers that have completed the third trimester visit, cortisol collection participation for at least one sample was moderately high (72%). Lastly, although the cortisol samples were taken before birth in the third trimester, no other cortisol levels were available earlier in pregnancy. Therefore, the current study was unable to assess whether birth weight is more sensitive to cortisol fluctuations in other prenatal exposure periods. It is also possible that cortisol levels during the first or second trimester could have had more effect on the fetus’s birth weight. If so, this study would have missed the effects of the biological stress response due to only having cortisol levels collected in the third trimester.

## 5. Conclusions

Within this study of predominately low-income Hispanic women, there were no significant associations of the maternal biological stress response through cortisol concentration (AUCg) and cortisol dynamics (DCS and CAR) during the third trimester of pregnancy with infant birth weight. Although not significant, an inverse relationship was seen between AUCg and birth weight while a less steep DCS was seen to increase birth weight. Assessing accurate cortisol patterns through salivary collection requires high compliance and motivation from study participants, which may be especially challenging populations facing unique stressors. More research is needed in larger study samples with more rigorous approaches of cortisol collection including more sampling days, while also considering methods to increase compliance, to better understand how prenatal stress and the associated biological stress response influences birth weight.

## Figures and Tables

**Table 1:** Descriptive statistics of 240 mother–infant dyads.

Characteristic	*N* (%) or Mean (SD)
**Maternal**	
Age (years)	29.1 (5.8)
Race	
White	206 (86.2%)
Black/African-American	24 (10.1%)
Asian	2 (0.8%)
American Indian/Alaskan Native	1 (0.4%)
>1 race	6 (2.5%)
Ethnicity	
Hispanic	195 (81.3%)
Non-Hispanic	45 (18.7%)
Highest Education Level	
Less than 12th grade	57 (23.8%)
High School	76 (31.7%)
Some college or technical school	59 (24.6%)
Complete 4 years of college	32 (13.3%)
Some graduate training	16 (6.6%)
Household Annual Income	
Less than $15,000	48 (20.0%)
$15,000–$29,999	65 (27.1%)
$30,000–$49,999	38 (15.8%)
$50,000–$99,999	13 (5.4%)
$100,000 or more	14 (5.8%)
Selected “Don’t Know”	62 (25.8%)
Pre-Pregnancy BMI (kg/m^2^)	29.1 (6.9)
**Infant**	
Female	131 (54.6%)
Birth weight (g)	3301.7 (497.3)
Low birth weight (<2500 g)	11 (4.6%)
Gestational age at birth (weeks)	39.0 (1.5)
Preterm birth (<37 weeks gestation)	23 (9.6%)
Birth Order	
First Born	85 (35.4%)
Second Born	68 (28.3%)
Third Born	41 (17.1%)
Fourth Born or more	37 (15.4%)
Unknown	9 (3.8%)
Type of Delivery	
Vaginal	179 (74.6%)
Cesarean section	61 (25.4%)

**Table 2:** Descriptive statistics of biological stress metrics.

Stress Metric	Mean (SD) *	Median *	Min-Max
Awakening Sample (nmol/L)	12.2 (7.7)	11.0	0.9–75.9
Awakening + 30 min Sample (nmol/L)	14.8 (9.6)	13.3	1.1–73.5
Afternoon Sample (nmol/L)	5.7 (4.8)	4.4	0.8–29.7
Bedtime Sample (nmol/L)	4.0 (4.4)	2.6	0.8–30.0
Area Under the Curve, grounded (AUCg)	116.7 (67.9)	99.9	13.3–424.9
Cortisol Awakening Response (CAR)	0.7 (1.8)	0.4	−5.7–9.8
Diurnal Cortisol Slope (DCS)	−0.6 (0.6)	−0.6	−4.7–1.1

* Mean (SD) and median provided due to variable skewness.

**Table 3:** Results of three independent models of biological stress measures (area under the curve, diurnal cortisol slope, cortisol awakening response) and infant birth weight.

Individual Biological Stress Metric	*β* (SE)	*p*
Area Under the Curve, grounded (AUCg) *^,1^	−5.6 (5.1)	0.25
Cortisol Awakening Response (CAR) *	−0.4 (15.3)	0.98
Diurnal Cortisol Slope (DCS) *	78.3 (44.9)	0.08

* Adjusted for gestational age at birth, parity, pre-pregnancy BMI, infant sex, maternal age, ethnicity, and study site of recruitment. ^1^ Beta coefficient was back transformed and is interpreted as the mean difference in birth weight associated with a 10% increase in the non-logged AUCg.

## Appendix A

**Table A1:** Results of three independent models of biological stress measures (Area Under the Curve, Diurnal Cortisol Slope, Cortisol Awakening Response) and infant birth weight in full-term births (≥37 weeks).

Individual Biological Stress Metric	β (SE)	*p*
Area Under the Curve, grounded (AUCg) *^,1^	−7.8 (5.6)	0.17
Cortisol Awakening Response (CAR) *	−1.7 (16.2)	0.91
Diurnal Cortisol Slope (DCS) *	87.1 (27.9)	0.07

* Adjusted for gestational age at birth, parity, pre-pregnancy BMI, infant sex, maternal age, ethnicity, and study site of recruitment. ^1^ Beta coefficient was back transformed and is interpreted as the mean difference in birth weight associated with a 10% increase in the non-logged AUCg.

## References

[1] Belbasis L., Savvidou M.D., Kanu C., Evangelou E., Tzoulaki I. (2016). Birth weight in relation to health and disease in later life: An umbrella review of systematic reviews and meta-analyses. BMC Med..

[2] Camerota M., Bollen K.A. (2016). Birth Weight, Birth Length, and Gestational Age as Indicators of Favorable Fetal Growth Conditions in a US Sample. PLoS ONE.

[3] Barker D.J., Hales C.N., Fall C.H., Osmond C., Phipps K., Clark P.M. (1993). Type 2 (non-insulin-dependent) diabetes mellitus, hypertension and hyperlipidaemia (syndrome X): Relation to reduced fetal growth. Diabetologia.

[4] Cutland C.L., Lackritz E.M., Mallett-Moore T., Bardají A., Chandrasekaran R., Lahariya C., Nisar M.I., Tapia M.D., Pathirana J., Kochhar S. (2017). Low birth weight: Case definition & guidelines for data collection, analysis, and presentation of maternal immunization safety data. Vaccine.

[5] Evensen E., Emaus N., Kokkvoll A., Wilsgaard T., Furberg A.S., Skeie G. (2017). The relation between birthweight, childhood body mass index, and overweight and obesity in late adolescence: A longitudinal cohort study from Norway, The Tromsø Study, Fit Futures. BMJ Open.

[6] Jornayvaz F.R., Vollenweider P., Bochud M., Mooser V., Waeber G., Marques-Vidal P. (2016). Low birth weight leads to obesity, diabetes and increased leptin levels in adults: The CoLaus study. Cardiovasc. Diabetol..

[7] Wang S.F., Shu L., Sheng J., Mu M., Wang S., Tao X.Y., Xu S.J., Tao F.B. (2014). Birth weight and risk of coronary heart disease in adults: A meta-analysis of prospective cohort studies. J. Dev. Orig. Health Dis..

[8] Pan X.F., Tang L., Lee A.H., Binns C., Yang C.X., Xu Z.P., Zhang J.L., Yang Y., Wang H., Sun X. (2019). Association between fetal macrosomia and risk of obesity in children under 3 years in Western China: A cohort study. World J. Pediatrics WJP.

[9] Sparano S., Ahrens W., De Henauw S., Marild S., Molnar D., Moreno L.A., Suling M., Tornaritis M., Veidebaum T., Siani A. (2013). Being macrosomic at birth is an independent predictor of overweight in children: Results from the IDEFICS study. Matern. Child Health J..

[10] Wang Y., Gao E., Wu J., Zhou J., Yang Q., Walker M.C., Mbikay M., Sigal R.J., Nair R.C., Wen S.W. (2009). Fetal macrosomia and adolescence obesity: Results from a longitudinal cohort study. Int. J. Obes..

[11] Kramer M.S. (2003). The epidemiology of adverse pregnancy outcomes: An overview. J. Nutr..

[12] Geronimus A.T. (1992). The weathering hypothesis and the health of African-American women and infants: Eidence and speculations. Ethn. Dis..

[13] Geronimus A.T. (1996). Black/white differences in the relationship of maternal age to birthweight: A population-based test of the weathering hypothesis. Soc. Sci. Med..

[14] McDonald S.W., Kingston D., Bayrampour H., Dolan S.M., Tough S.C. (2014). Cumulative psychosocial stress, coping resources, and preterm birth. Arch. Women’s Ment. Health.

[15] Rondó P.H., Ferreira R.F., Nogueira F., Ribeiro M.C., Lobert H., Artes R. (2003). Maternal psychological stress and distress as predictors of low birth weight, prematurity and intrauterine growth retardation. Eur. J. Clin. Nutr..

[16] Staneva A., Bogossian F., Pritchard M., Wittkowski A. (2015). The effects of maternal depression, anxiety, and perceived stress during pregnancy on preterm birth: A systematic review. Women Birth J. Aust. Coll. Midwives.

[17] Riley W.J. (2012). Health disparities: Gaps in access, quality and affordability of medical care. Trans. Am. Clin. Climatol. Assoc..

[18] Nagahawatte N.T., Goldenberg R.L. (2008). Poverty, maternal health, and adverse pregnancy outcomes. Ann. N. Y. Acad. Sci..

[19] Martin J.A., Hamilton B.E., Osterman M.J.K., Driscoll A.K., Drake P. (2018). Births: Final Data for 2017. Natl. Vital Stat. Rep. Cent. Dis. Control Prev. Natl. Cent. Health Stat. Natl. Vital Stat. Syst..

[20] Arbona C., Olvera N., Rodriguez N., Hagan J., Linares A., Wiesner M. (2010). Acculturative Stress Among Documented and Undocumented Latino Immigrants in the United States. Hisp. J. Behav. Sci..

[21] Velasco-Mondragon E., Jimenez A., Palladino-Davis A.G., Davis D., Escamilla-Cejudo J.A. (2016). Hispanic health in the USA: A scoping review of the literature. Public Health Rev..

[22] Adam E.K., Kumari M. (2009). Assessing salivary cortisol in large-scale, epidemiological research. Psychoneuroendocrinology.

[23] Fries E., Dettenborn L., Kirschbaum C. (2009). The cortisol awakening response (CAR): Facts and future directions. Int. J. Psychophysiol. Off. J. Int. Organ. Psychophysiol..

[24] Pruessner J.C., Wolf O.T., Hellhammer D.H., Buske-Kirschbaum A., von Auer K., Jobst S., Kaspers F., Kirschbaum C. (1997). Free cortisol levels after awakening: A reliable biological marker for the assessment of adrenocortical activity. Life Sci..

[25] Allolio B., Hoffmann J., Linton E.A., Winkelmann W., Kusche M., Schulte H.M. (1990). Diurnal salivary cortisol patterns during pregnancy and after delivery: Relationship to plasma corticotrophin-releasing-hormone. Clin. Endocrinol..

[26] Jung C., Ho J.T., Torpy D.J., Rogers A., Doogue M., Lewis J.G., Czajko R.J., Inder W.J. (2011). A longitudinal study of plasma and urinary cortisol in pregnancy and postpartum. J. Clin. Endocrinol. Metab..

[27] Gudmundsson A., Carnes M. (1997). Pulsatile adrenocorticotropic hormone: An overview. Biol. Psychiatry.

[28] Johnson M.L., Virostko A., Veldhuis J.D., Evans W.S. (2004). Deconvolution analysis as a hormone pulse-detection algorithm. Methods Enzymol..

[29] Bloom S.L., Sheffield J.S., McIntire D.D., Leveno K.J. (2001). Antenatal dexamethasone and decreased birth weight. Obstet. Gynecol..

[30] Entringer S., Buss C., Wadhwa P.D. (2012). Prenatal stress, telomere biology, and fetal programming of health and disease risk. Sci. Signal..

[31] French N.P., Hagan R., Evans S.F., Godfrey M., Newnham J.P. (1999). Repeated antenatal corticosteroids: Size at birth and subsequent development. Am. J. Obstet. Gynecol..

[32] Reynolds R.M. (2013). Glucocorticoid excess and the developmental origins of disease: Two decades of testing the hypothesis--2012 Curt Richter Award Winner. Psychoneuroendocrinology.

[33] Togher K.L., Togher K.L., O’Keeffe M.M., O’Keeffe M.M., Khashan A.S., Khashan A.S., Gutierrez H., Gutierrez H., Kenny L.C., Kenny L.C. (2014). Epigenetic regulation of the placental HSD11B2 barrier and its role as a critical regulator of fetal development. Epigenetics.

[34] Crowther C.A., Middleton P.F., Voysey M., Askie L., Zhang S., Martlow T.K., Aghajafari F., Asztalos E.V., Brocklehurst P., Dutta S. (2019). Effects of repeat prenatal corticosteroids given to women at risk of preterm birth: An individual participant data meta-analysis. PLoS Med..

[35] Bolten M.I., Wurmser H., Buske-Kirschbaum A., Papoušek M., Pirke K.M., Hellhammer D. (2011). Cortisol levels in pregnancy as a psychobiological predictor for birth weight. Arch. Women‘s Ment. Health.

[36] Braithwaite E.C., Hill J., Pickles A., Glover V., O′Donnell K., Sharp H. (2018). Associations between maternal prenatal cortisol and fetal growth are specific to infant sex: Findings from the Wirral Child Health and Development Study. J. Dev. Orig. Health Dis..

[37] D′Anna-Hernandez K.L., Hoffman M.C., Zerbe G.O., Coussons-Read M., Ross R.G., Laudenslager M.L. (2012). Acculturation, maternal cortisol, and birth outcomes in women of Mexican descent. Psychosom. Med..

[38] Giesbrecht G.F., Campbell T., Letourneau N. (2015). Sexually dimorphic adaptations in basal maternal stress physiology during pregnancy and implications for fetal development. Psychoneuroendocrinology.

[39] Gilles M., Otto H., Wolf I.A.C., Scharnholz B., Peus V., Schredl M., Sütterlin M.W., Witt S.H., Rietschel M., Laucht M. (2018). Maternal hypothalamus-pituitary-adrenal (HPA) system activity and stress during pregnancy: Effects on gestational age and infant’s anthropometric measures at birth. Psychoneuroendocrinology.

[40] Guardino C.M., Schetter C.D., Saxbe D.E., Adam E.K., Ramey S.L., Shalowitz M.U. (2016). Diurnal salivary cortisol patterns prior to pregnancy predict infant birth weight. Health Psychol. Off. J. Div. Health Psychol. Am. Psychol. Assoc..

[41] Hompes T., Vrieze E., Fieuws S., Simons A., Jaspers L., Van Bussel J., Schops G., Gellens E., Van Bree R., Verhaeghe J. (2012). The influence of maternal cortisol and emotional state during pregnancy on fetal intrauterine growth. Pediatric Res..

[42] Kivlighan K.T., DiPietro J.A., Costigan K.A., Laudenslager M.L. (2008). Diurnal rhythm of cortisol during late pregnancy: Associations with maternal psychological well-being and fetal growth. Psychoneuroendocrinology.

[43] Spicer J., Werner E., Zhao Y., Choi C.W., Lopez-Pintado S., Feng T., Altemus M., Gyamfi C., Monk C. (2013). Ambulatory assessments of psychological and peripheral stress-markers predict birth outcomes in teen pregnancy. J. Psychosom. Res..

[44] Bublitz M.H., Bourjeily G., D’Angelo C., Stroud L.R. (2018). Maternal Sleep Quality and Diurnal Cortisol Regulation over Pregnancy. Behav. Sleep Med..

[45] Bublitz M.H., Vergara-Lopez C., O’Reilly Treter M., Stroud L.R. (2016). Association of Lower Socioeconomic Position in Pregnancy with Lower Diurnal Cortisol Production and Lower Birthweight in Male Infants. Clin. Ther..

[46] Smew A.I., Hedman A.M., Chiesa F., Ullemar V., Andolf E., Pershagen G., Almqvist C. (2018). Limited association between markers of stress during pregnancy and fetal growth in ‘Born into Life’, a new prospective birth cohort. Acta Paediatrica.

[47] Cherak S.J., Giesbrecht G.F., Metcalfe A., Ronksley P.E., Malebranche M.E. (2018). The effect of gestational period on the association between maternal prenatal salivary cortisol and birth weight: A systematic review and meta-analysis. Psychoneuroendocrinology.

[48] Adam E.K., Quinn M.E., Tavernier R., McQuillan M.T., Dahlke K.A., Gilbert K.E. (2017). Diurnal cortisol slopes and mental and physical health outcomes: A systematic review and meta-analysis. Psychoneuroendocrinology.

[49] Stalder T., Kirschbaum C., Kudielka B.M., Adam E.K., Pruessner J.C., Wüst S., Dockray S., Smyth N., Evans P., Hellhammer D.H. (2016). Assessment of the cortisol awakening response: Expert consensus guidelines. Psychoneuroendocrinology.

[50] Bastain T.M., Chavez T., Habre R., Girguis M.S., Grubbs B., Toledo-Corral C., Amadeus M., Farzan S.F., Al-Marayati L., Lerner D. (2019). Study Design, Protocol and Profile of the Maternal And Developmental Risks from Environmental and Social Stressors (MADRES) Pregnancy Cohort: A Prospective Cohort Study in Predominantly Low-Income Hispanic Women in Urban Los Angeles. BMC Pregnancy Childbirth.

[51] Pruessner J.C., Kirschbaum C., Meinlschmid G., Hellhammer D.H. (2003). Two formulas for computation of the area under the curve represent measures of total hormone concentration versus time-dependent change. Psychoneuroendocrinology.

[52] Clow A., Hucklebridge F., Stalder T., Evans P., Thorn L. (2010). The cortisol awakening response: More than a measure of HPA axis function. Neurosci. Biobehav. Rev..

[53] Rotenberg S., McGrath J.J., Roy-Gagnon M.H., Tu M.T. (2012). Stability of the diurnal cortisol profile in children and adolescents. Psychoneuroendocrinology.

[54] Committee on Obstetric Practice, American Institute of Ultrasound in Medicine, & Society for Maternal-Fetal Medicine (2017). Committee Opinion No 700: Methods for Estimating the Due Date. Obstet. Gynecol..

[55] Koren G., Boskovic R., Hard M., Maltepe C., Navioz Y., Einarson A. (2002). Motherisk-PUQE (pregnancy-unique quantification of emesis and nausea) scoring system for nausea and vomiting of pregnancy. Am. J. Obstet. Gynecol..

[56] Nijm J., Kristenson M., Olsson A.G., Jonasson L. (2007). Impaired cortisol response to acute stressors in patients with coronary disease. Implications for inflammatory activity. J. Intern. Med..

[57] Saxbe D. (2008). A field (researcher’s) guide to cortisol: Tracking HPA axis functioning in everyday life. Health Psychol. Rev..

